# Degradation Mechanism Detection in Photovoltaic Backsheets by Fully Convolutional Neural Network

**DOI:** 10.1038/s41598-019-52550-6

**Published:** 2019-11-06

**Authors:** Binbin Zhang, Joydan Grant, Laura S. Bruckman, Olga Wodo, Rahul Rai

**Affiliations:** 1 Mechanical and Aerospace Engineering Department, University at Buffalo, Buffalo, USA; 2 Mechanical Engineering Tuskegee University, Tuskegee, AL USA; 3 Materials Design and Innovation Department, University at Buffalo, Buffalo, USA; 4 Department of Materials Science and Engineering, Case Western Reserve University, Cleveland, OH USA

**Keywords:** Computational methods, Computer science

## Abstract

Materials and devices age with time. Material aging and degradation has important implications for lifetime performance of materials and systems. While consensus exists that materials should be studied and designed for degradation, materials inspection during operation is typically performed manually by technicians. The manual inspection makes studies prone to errors and uncertainties due to human subjectivity. In this work, we focus on automating the process of degradation mechanism detection through the use of a fully convolutional deep neural network architecture (F-CNN). We demonstrate that F-CNN architecture allows for automated inspection of cracks in polymer backsheets from photovoltaic (PV) modules. The developed F-CNN architecture enabled an end-to-end semantic inspection of the PV module backsheets by applying a contracting path of convolutional blocks (encoders) followed by an expansive path of decoding blocks (decoders). First, the hierarchy of contextual features is learned from the input images by encoders. Next, these features are reconstructed to the pixel-level prediction of the input by decoders. The structure of the encoder and the decoder networks are thoroughly investigated for the multi-class pixel-level degradation type prediction for PV module backsheets. The developed F-CNN framework is validated by reporting degradation type prediction accuracy for the pixel level prediction at the level of 92.8%.

## Introduction

Photovoltaic (PV) energy has been growing in the global energy market and will continue to grow with an increase in durability and reliability and the subsequent reduction in cost^1^. PVs modules typically have a three-layer polymer laminate as the backsheet of the module to provide an environmental protective barrier for the PV cells. Additionally, the backsheet provides safety for the module due to the high dielectric breakdown strength of polyethylene terephthalate (PET), which is often used as a core layer in backsheets^2^. PV backsheets are susceptible to degradation from environmental stressors such as irradiance, temperature, pollution, and humidity^3–6^. Various degradation mechanisms, such as delamination and crack formation in PV module backsheets, leads to the failure of the safety and environmental barrier provided by the backsheet requiring repair or replacement^7–9^. The fundamental understanding of underlying degradation mechanisms and pathways is largely missing and hinders the ability to predict the lifetime of PV module backsheets.

Currently, manual field surveys by technicians has been the main method to identify backsheet degradation and failure^10,11^. Automated field surveys have emerged as an alternative for large scale commercial PV sites. These field surveys include techniques such as fluorescence, electroluminescence, photoluminescence, visual, and infrared imaging for the analysis of cell cracking, corrosion, and encapsulant degradation^12–15^. Although approaches to collect the data exists (e.g., via drones, airplanes, or by instruments that can be moved along racks), the progress has not been paralleled by the data analytic tools to automate degradation detection and analysis. Additionally, there has been little focus on automated methods to detect failure or degradation on the backsheets. Such an automated way to identify degradation mechanism would reduce the operation and maintenance of PV farms and provide insights into the degradation mechanisms of fielded backsheets. More importantly, a large amount of data collected and annotated during operation would accelerate the rate of establishing a reliable relationship between various environmental conditions and degradation rates of materials^10,16,17^.

The progress has been made in other applications, where various signals have also leveraged to detect defects in materials and structures. For example in steel beams, cracks have been identified from vibrational changes by leveraging the wavelet analysis^18^. Mechanical impedance^19^ and ultrasounds have been also used to detect the internal defects^20^. The progress has been also made in the area of machine vision for defect detection^21–24^. For example, Koch *et al*.^22^ investigated decision trees and support vector machines (SVM) methods for the task of defect detection in concrete and asphalt civil infrastructure. In that approach, the fixed rules are used to select a subset of regions in the image for which handcrafted features are computed. However, handcrafted features require significant domain knowledge, effort, and often fine-tuning to adjust them to perform efficiently in a particular scenario. The alternative approach involves an automated feature development and is considered the key advantage of deep learning based approaches. These approaches learn discriminative representations from the data without the need for handcrafted features. The learned representations offer high effectiveness to perform the mapping between automated features and the output of interest. At the same time, it has been shown that the learned representation might be difficult to notice or deduce for domain experts or conventional supervised learning methods.

It is the unremitting success of deep learning techniques in image classification and object detection tasks that motivated researchers to explore the capabilities of such network for pixel-level labeling tasks, such as scene labeling^25,26^ and semantic segmentation^27–29^. Many different models have been proposed for semantic segmentation^30–33^. The most successful state-of-art deep learning techniques for semantic segmentation spring from a common breakthrough: the fully convolutional neural network (F-CNN) by Long *et al*.^29^. This network is trained to learn hierarchies of features. The learned features are fused to achieve a non-linear, local-to-global feature representation that enables a pixel-wise inference. The F-CNN framework has shown a significant improvement in the segmentation accuracy over traditional methods on standard datasets like PASCAL VOC benchmark^34^.

Motivated by the excellent performance of F-CNN on segmentation tasks reported in the literature and inspired by the ideas of flexibility in segmentation networks, we adapted the F-CNN for the task of degradation type detection of PV backsheets. Specifically, we have customized a F-CNN for degradation mechanism type detection by altering the standard feature extraction and expansion structure of the F-CNN to improve the accuracy of results on PVs degradation mechanism type detection. While previous work has attempted to address vision problems generally, this paper is concerned with the development of a semantic segmentation method that can be used for automated PV module degradation mechanism detection, e.g., crack inspection. In this paper, we outline the essential system components of an automated inspection system of PVs. The automated computer vision is based upon a deep learning architecture that falls under the family of fully convolutional neural networks (F-CNNs). Validation test demonstrates the high speed and accuracy of the proposed F-CNN architecture. Our approach and networks are generic and can be used for segmentation and identification of other cracks types and inspection of other material systems.

## Results and Discussion

**Figure 1:**
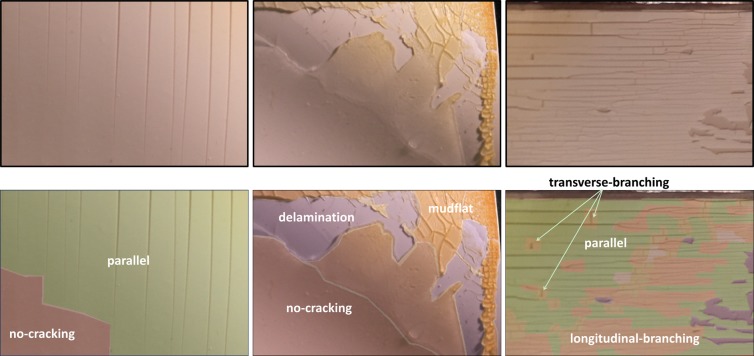
Examples of representative PVs polymer backsheets with different categories of crack patterns: parallel cracks, mudflat, parallel cracks, transverse-branching, and longitudinal-branching cracks. Three original representative images are shown in the top row. Labelled and annotated images are repeated in the bottom row.

In this work, the detection of degradation modes in backsheets is discussed. Different types of surface patterns are observed in PV module backsheet films exposed to accelerated and real-world exposures (Klinke, *et al*.^35^). The all the degradation types are observed on the inner-layer (i.e., the sun-side layer in a PV module), which was not directly exposed to irradiance. Fig. 1 depicts three representative images the various categories of degradation. The observed patterns can be mainly grouped into six categories: no-cracking, parallel, delamination, transverse-branching, longitudinal-branching, and mudflat. The no-cracking region refers to the region with the absence of any types of cracks. The no-cracking regions are desirable, but do not belong to any defect mechanism pattern. The parallel cracks in Fig. 1 are oriented parallel in the vertical direction (or along Y-axis of the image). The large-scale loss of adhesion leads to pieces of the inner layer to fall off and in turn leads to the delamination regions as shown in Fig. 1. The transverse-branching cracks are crack patterns that are perpendicular to parallel cracks (horizontal direction or along the x-axis). If there are branches on the parallel cracks and the branches are in the same direction with the parallel cracks, the type of branch crack is annotated as longitudinal-branching. Finally, the cracks are labeled as mudflat cracks when the branches of the cracks are oriented in multiple directions. The examples of transverse-branching, longitudinal-branching, and mudflat are also shown in Fig. 1.

The data set consists of 34 varying resolution images of the inner-layer (sun side) of backsheet films exposed to two accelerated exposures with eight steps of 500 h and two real-world exposures with six steps of 2 months^35^. Samples were exposed with the air side of the backsheet films facing the irradiance source in two different ASTM G154-04 cycle four exposures^36^ using UVA-340 fluorescent ultraviolet lamps (wavelengths 280–400 nm) in Q-Lab QUV accelerated weathering testers. One exposure was a cyclic exposure of 8 h of 1.55 W/m^2^/nm at a 70 °C chamber temperature followed by 4 h of darkness and condensing humidity at 50 °C and the other without the dark condensing humidity step. Real-world exposures were conducted in Cleveland, Ohio between July 2, 2013, and October 7, 2014, on two-axis trackers in sample trays with and without irradiance concentration. The images were collected using PAX-it PAXcam camera in a photo lightbox. These samples and exposures are described in detail in Klinke *et al*.^35^.

The PV image dataset is annotated by human experts from Case Western Reserve University. Specifically, all images in the dataset are labeled manually. The image annotation tool LabelMe^37^ was used for labeling purposes. The tool allows users to annotate a class by clicking along the boundary of the desired class and indicating its identity. In Fig. 1, the raw dataset images are depicted in the first row. The second-row images correspond to the manually annotated images of various crack regions using LabelMe. The annotated images are considered as ground truth labels of the raw images. As a result of annotation, the six categories are identified. The categories include no-cracking, parallel, delamination, transverse-branching, longitudinal-branching, and mudflat. These categories and are encoded as class labels 1, 2, 3, 4, 5, and 6, respectively. The remaining region of the image (not belonging to the first six classes) is considered as a background and assigned a different class category (class 0). Therefore, there are in total of seven classes (*N* = 7). The images obtained from the backsheet film study are of different resolution. Furthermore, the initial dataset size of 34 images may be insufficient to train the F-CNN model. Therefore, the initial sets of annotated images are split into image blocks. Examples of the image block are depicted in the first row of Fig. 1. Following this strategy, the initial set of 34 images is processed to generate 286 image block samples. Each image block sample is 320 pixels wide and 480 pixels high (320 × 480). The 286 image block samples are considered the input dataset, *I*, for the analysis. For training and evaluation of the model, the dataset *I* is shuffled and randomly split into three non-overlapping sets, namely a training set *I*_*Train*_ (170 examples), a validation set *I*_*Vald*_ (73 examples), and a test set *I*_*Test*_ (43 examples), respectively. To avoid variations between the training set and the test set, the label-preserving transformation was applied after splitting, specifically horizontal and vertical mirroring for the training set for inflating the size of the training dataset.

The annotated dataset is used to train the F-CNN networks. The architecture of the final F-CNN is determined through empirical studies, as generic design rules for constructing CNN are still elusive. Specifically, our architecture is developed by varying the encoding and decoding configuration. In this sense, this paper makes two contributions. First, several architectures of F-CNN are investigated. Next, the accuracy of the final architecture is discussed in the context of the application of interest. In the next two subsections, the corresponding results are discussed.

### Design of F-CNN for detection of backsheet degradation types

**Figure 2:**
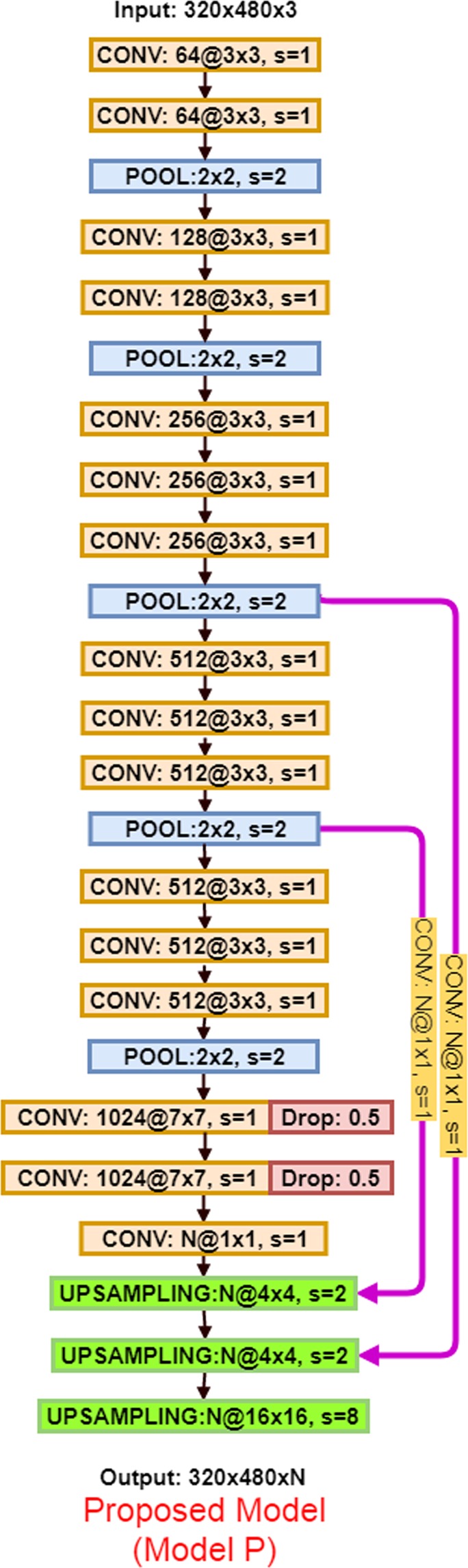
Overview of the F-CNN model for autonomous recognition of degradation mechanism.

The proposed network consists of an encoding part and a decoding part. In the encoding part, high level abstract features maps or representations are extracted from input images. The extraction is achieved through applying a series of convolutional and pooling layers. In the decoding part, the abstract features are gradually reconstructed to the pixel-level prediction of the input images. The reconstruction is accomplished through relaying the intermittent feature representations from encoding part to decoding part through concatenation layers. The network architecture of the F-CNN for degradation mechanism detection in backsheets is illustrated in Fig. 2. Two structures of the F-CNN architecture: encoder and decoder are detailed below. Table 1 summarizes a nomenclature used.

**Table 1:** Nomenclature used to describe the network architecture and parameters used during the experiments.

	Nomenclature
CONV: *n*_*f*_ @ *n*_*x*_ × *n*_*y*_, *s* = *D*	Convolution layer with the number of filters *n*_*f*_, filter size *n*_*x*_ × *n*_*y*_, and stride *D* used
POOL: *n*_*p*_ × *n*_*p*_, *s* = *D*	Max pooling layer with filter size *n*_*p*_ × *n*_*p*_ and stride *D*
Drop: *λ*	Dropout, with drop probability *λ*
EPOCH	Number of passes over the dataset during training
LR	Learning rate used during training
UPSAMPLING: *n*_*f*_ @ *n*_*x*_ × *n*_*y*_, *s* = *D*	Upsampling layer with the number of filters *n*_*f*_, filter size *n*_*x*_ × *n*_*y*_, and stride *D* used
*N*	Total number of classes
	Feature fusion

#### Encoder structures

The convolutional layer (CONV layer) is the basic building block of a deep neural network model. The CONV layer performs two-dimensional convolution of the input image using a set of filters *W*, generating a set of feature maps *h*. Mathematically, the operation is expressed as follows:

$$h=W\ast X+b$$

where *b* denotes the bias of the filter, and ‘*’ represents the convolution operation.

Activation function (ReLU layer): A non-linear activation function handles the non-linearities of the mapping between input and output. In general, the Rectified Linear Unit (*ReLU*(*h*) = *max* (0, *h*)) is used as the neuron activation function, as it performs well with respect to runtime and generation error^38^. This function is added after each convolutions layer.

Pooling layer (POOL layer): The POOL layer receives feature maps and resizes them into smaller maps. The most favorable POOL layer choice is max-pooling, where each map is subsampled with the maximum value over *n*_*p*_ × *n*_*p*_ adjacent regions. Max-pooling is performed as it introduces small invariance to translation and distortion and leads to faster convergence and better generalization^39,40^.

#### Decoder structures

**Figure 3:**
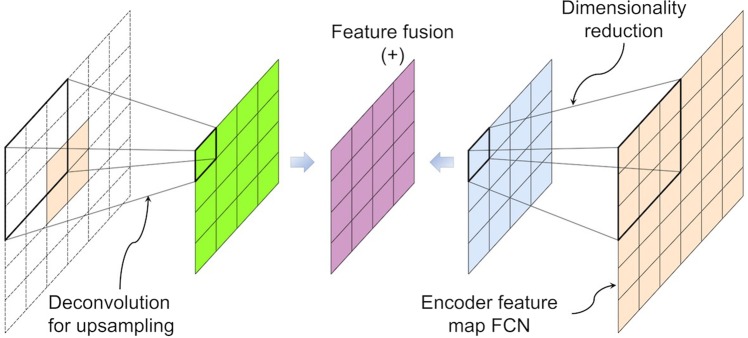
Upsampling layer and feature fusion.

Upsampling layer (UPSAMPLING): Upsampling is a procedure to connect coarse outputs to dense pixels through interpolation. F-CNN-based architectures make use of learnable upsampling filters to upsample feature maps. The upsampling kernels are learned through the usage of transposed convolution (deconvolution)^41^, in which zero paddings and stride are specified to increase the size of feature maps instead. Figure 3 illustrates the upsampling process through the deconvolutional layer.

Feature fusion: Fusion is an element of F-CNN that enables the addition of context information to a fully convolutional architecture. As demonstrated in Fig. 3, the upsampled feature maps generated by the deconvolutional layer are added elementwise to the corresponding feature maps generated by the convolutional layer in the encoder.

There is no unique approach to design the encoder and decoder architectures. In this paper, various designs are explored to deliver the highest validation accuracy and yield low generalization error for new data. Regardless of the details of the architecture, the parameters of the network are determined via the training process.

#### Training process

Once the architecture is decided, the final step is to train the model using the dataset. In this paper, the aim is to map the input image to the set of classes. Specifically, the goal is to determine the complex end-to-end mapping function that transforms the input image from measurement {*X*_*i*_} to its corresponding multi-class image {*Y*_*i*_}. The output multi-class image consists of pixels annotated with the degradation category (0–6).

The network parameters are iteratively updated using backpropagation^42,43^ to minimize the loss. The categorical cross-entropy loss^44^ is applied for evaluating the output error. The output error is obtained by computing the deviation (error) of the network outcome *Y*_*i*_ with the desired ground-truth $\{{Y^{\prime} }_{i}\}$. The cross-entropy loss is defined as:

$$Loss=-\,\sum \,{Y^{\prime} }_{i}\,\log \,({Y}_{i})$$

where *Y*_*i*_ is a function of the input image {*X*_*i*_} and the network parameters (i.e., *W* and *b*). The *Adam* optimization method is used^45^ as it offers faster convergence than the standard stochastic gradient descent method.

The training process heavily depends on the amount of data available. In many materials science problems, the cost of generating data is high, and various strategies are needed to address this issue. In our problem, there are two significant challenges. First of all, we augment our dataset by splitting the collected datasets into the image blocks as described in the introduction of the Results Section. This operation allowed to increase the size of the dataset by the factor of 8. Another challenge stems from the unbalanced data towards some classes of cracks. In our dataset, seven classes are represented by significantly different numbers of pixels per region. Finally, whenever the data is limited the overfitting may occur hampering the generalizability of the F-CNN. The details of two strategies to address the above issues are detailed below.

#### Data balancing strategy

The unbalanced data used in the training can cause the learning algorithm to become biased towards the dominating class^46^. In order to balance the different class frequencies and thus their contribution to the loss function, we introduce weighting coefficients *η* for each semantic class. The coefficient is defined as:

$${\alpha }_{i}=\frac{{\sum }_{i=0}^{N-1}{p}_{i}}{{p}_{i}}$$

$${\eta }_{i}=\frac{{\alpha }_{i}}{{\sum }_{i=0}^{N-1}{\alpha }_{i}}$$

where *p*_*i*_ is the number of pixels belong to class *i* in the training set, and ${\sum }_{i=0}^{N-1}\,{p}_{i}$ is the total pixel count over all classes. The loss function is updated accordingly:

$$Los{s}_{weighted}=\eta \times Loss$$

In this way, the importance of sparse classes (in terms of the pixel areas) is corrected.

#### Regularization strategy

Our network architecture is relatively deep, and the availability of data is limited, regularization needs to be used to mitigate the generalization test error of the algorithm^47^. Among the variety of regularization techniques available, we applied L2 regularization and dropout. L2 regularization applies a penalty on large network parameters and forces them to be relatively small^48^. Dropout refers to a technique where a fraction of randomly selected activations are ignored during training. It helps to reduce overfitting by not allowing the model to be heavily dependent on the output of one or a few neurons. According to Srivastava *et al*.^49^, Gaussian dropout could perform better than the classical Bernoulli dropout. The use of Gaussian dropout equivalent to adding a Gaussian distributed random variable with zero mean and standard deviation. The Gaussian dropout is defined as follows:

$$Drop=\sqrt{\frac{\lambda }{1-\lambda }},\lambda \in (0,1)$$

where *λ* is the drop probability.

In our proposed architectures, we utilized L2 regularization and Gaussian dropout regularization strategies. The L2 regularization is applied after each activation function. Dropout is added after the last two convolution layers. As shown in Fig. 2, *λ* = 0.5 is added after the convolutional layers which have 1024 kernels.

#### Evaluation strategy

To assess the performance of different architectures, we computed several metrics^50^:

1. *Pixel Accuracy*: it is a metric computing a ratio between the amount of correctly classified pixels and the total number of pixels.
  $$pixelAcc=\frac{{\sum }_{i=0}^{N-1}{p}_{ii}}{{\sum }_{i=0}^{N-1}{\sum }_{j=0}^{N-1}{p}_{ij}}$$
2. *Mean Intersection over Union* (*meanIU*): it measures the intersection over the union of the labeled segments for each class and reports the average. It computes the ratio between the number of true positives (intersection) and the sum of true positives, false negatives, and false positives (union).
  $$meanIU=\frac{1}{N}\mathop{\sum }\limits_{i=0}^{N-1}\frac{{p}_{ii}}{{\sum }_{j=0}^{N-1}{p}_{ij}+{\sum }_{j=0}^{N-1}{p}_{ij}-{p}_{ii}}$$
3. *Per-class accuracy*: this is simply the proportion of correctly labeled pixels on a per-class basis. The $perClassAcc$ for class $i$ is defined as:

$$perClassAc{c}_{i}=\frac{{p}_{ii}}{{\sum }_{j=0}^{N-1}{p}_{ij}}$$

where *N* is the number of classes, *p*_*ij*_ is the number of pixels of class *i* inferred to belong to class *j*, *p*_*ii*_ represents true positives (the number of pixels correctly classified), *p*_*ij*_ represents false positives (the number of pixels incorrectly classified) and *p*_*ji*_ represents false negatives (the number of pixels which are wrongly not classified), respectively.

#### Empirical studies to identify the F-CNN architecture

**Figure 4:**
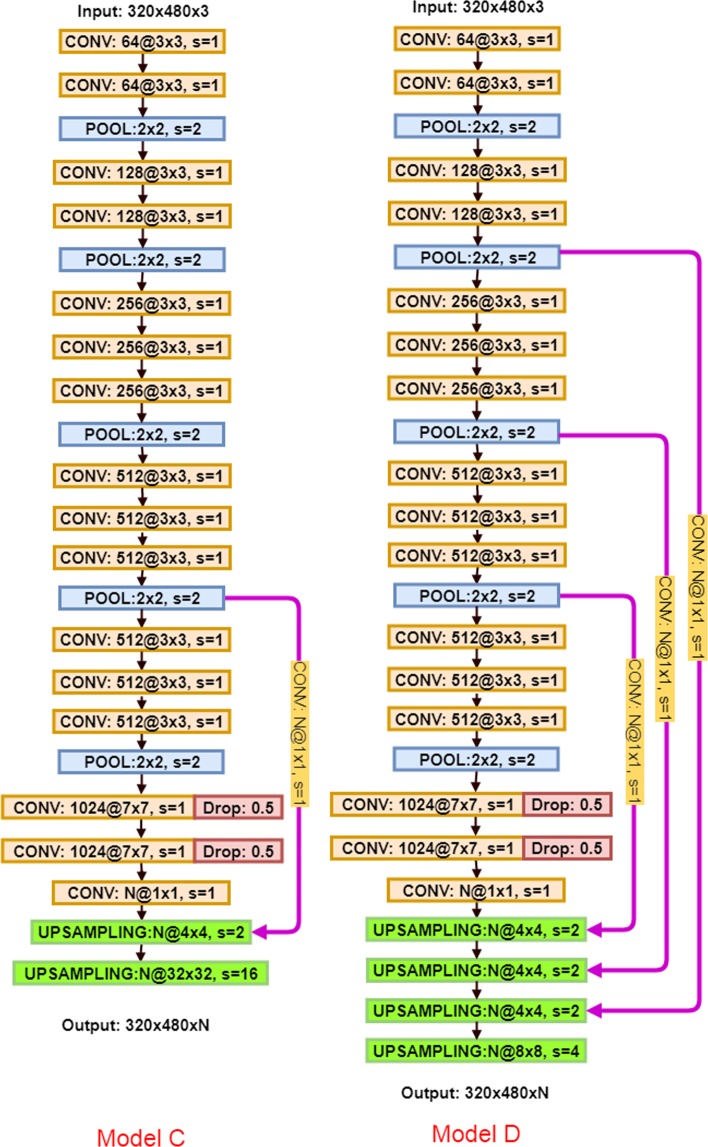
Model A and Model B architectures.

In this paper, we study different encoder and decoder architectures to identify the final architecture which results in the highest validation accuracy and yields low generalization error for new data. We vary details of encoder and decoder structure independently. We first investigate the encoder structure as it plays a crucial role in learning distinctive features from the input dataset. Encoder structure has a strong effect on the computational performance of F-CNN. To develop an encoder architecture on the task of crack inspection, we evaluated the proposed F-CNN model P and two other models. The architecture of the two models (Model A and B) are as shown in Fig. 4. In the three different architectures, the number of CONV layers were changed. Models A, B, and P used 6, 13, and 16 layers of CONV, respectively. The last convolution layer is added to facilitate the prediction of the decoder to the *N* categories.

**Figure 5:**
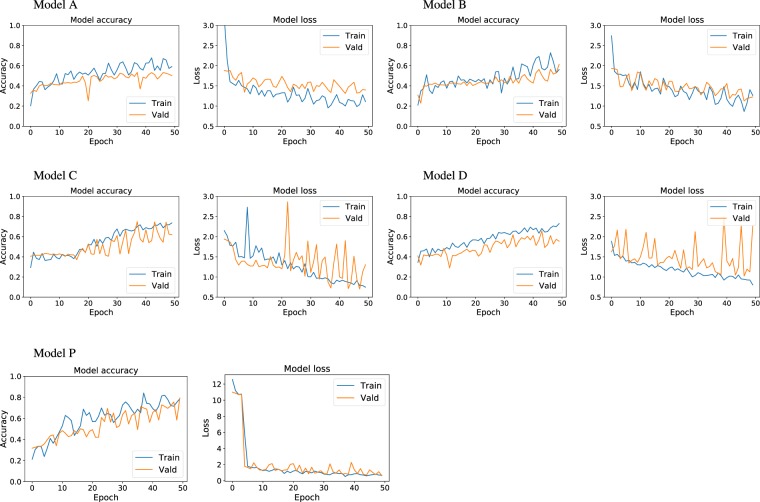
Accuracy and loss plots as a function of epochs for several models.

The accuracy and loss plots for model A, B, and proposed Model P are presented in Fig. 5. The evaluation results on test data are listed in Table 2. The time shown in the table is the training and validation time. It was observed that the test accuracy is better for complex models. A possible reason for the better performance of the complex model could be attributed to the complexity of features. As the complexity of crack features increases, the encoder structure needs to include more number of convolutional layers to extract the abstract contextual features from input images for the following pixel-level prediction. It is worth noting that as the accuracy increases, the computing time also increases since more number of parameters are trained in the system. Therefore, we did not enhance the complexity of the model beyond this design. The accuracy and loss plots also demonstrate that the training and validation accuracy of Model P climbs faster than Model A and B. Thus, the Model P was chosen for further improvement of our system.

**Table 2:** Performance metric for five investigated architectures. Three accuracy metrics were measured after 50 epochs with learning rate *LR* = 0.001.

Model	Pixel Accuracy	Mean IU	Time (h:m:s)
A	45.5%	17.8%	6:47:56
B	62.6%	20.2%	10:58:38
C	61.0%	22.4%	23:38:36
D	53.3%	21.5%	25:01:49
P	79.6%	49.7%	15:28:10

#### Decoder structure development

**Figure 6:**
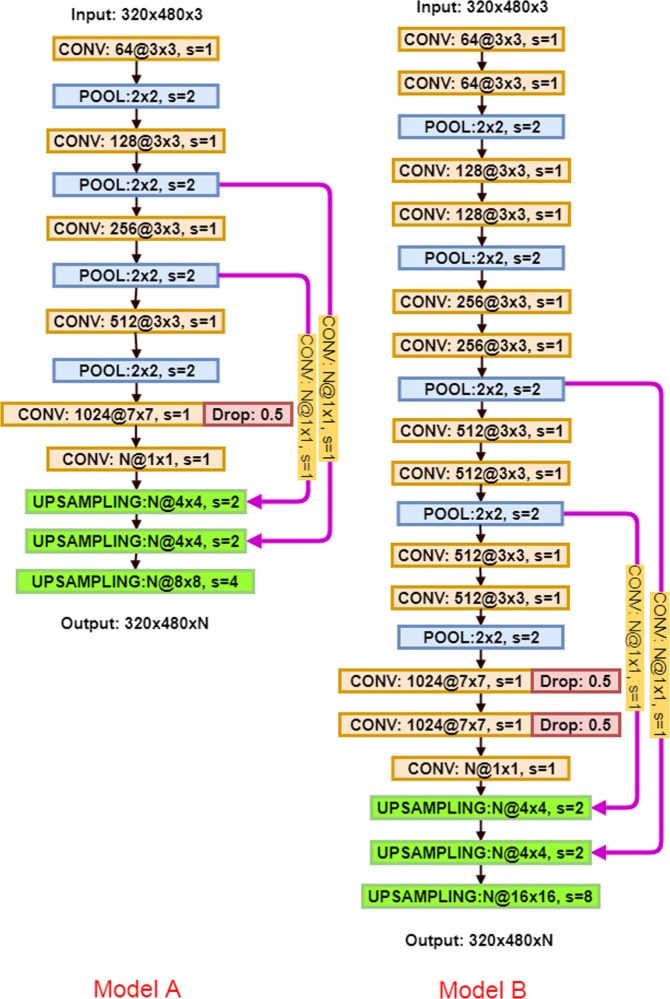
Model C and Model D architectures.

Apart from the importance of encoder part of the architecture which produces low-resolution image representations or feature maps, the role of decoder part is also significant as it maps those low-resolution images to the pixel-wise predictions for segmentation. Two more different decoder structures, i.e., Model C and D in Fig. 6, are investigated in our experiment. The variation between Model C, D, and our proposed Model P is on the number of upsampling layers and feature fusion times. In model C, we upsampled the last convolution layer, fused the feature information with the fourth pooling payer feature maps, and then upsampled to the size of the input image for pixel prediction. While in model D, more intermittent feature representations from encoding part learned from the input images are concatenated into the decoding part for inference. The accuracy and loss plots for training and validation dataset are presented in Fig. 5. The test result is listed in Table 2. Our results indicate that Model P is predominant in both the test accuracy and training time. Therefore, model P is chosen as our final model.

The above studies resulted in the final architecture of F-CNN shown in Fig. 2. The F-CNN contains 16 stacked convolution layers and two feature fusion and three upsampling layers. Each convolution layer is followed by ReLU activation function. The architecture has roughly 41.4 million parameters to be estimated. Such a high-dimensional model is prone to overfitting taking into account the relatively small datasets under consideration. To mitigate overfitting, we applied data augmentation during data preparation, L2 regularization after each convolution layer and Gaussian dropout on last two convolution layers during training. Finally, in the decoding part, we fused the feature information extracted from the last convolution layer with feature maps obtained after the third and fourth pooling layer for image prediction.

**Figure 7:**
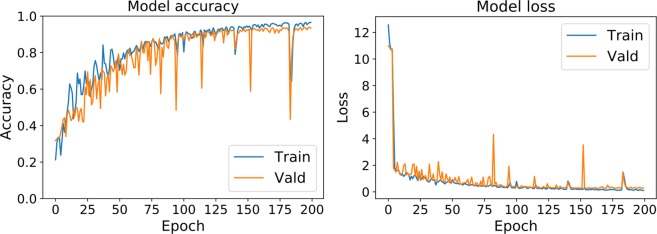
The accuracy and loss plots for our proposed Model P.

The final architecture is evaluated for 200 epochs to obtain an estimate of its generalization performance. The run time for the training process and 200 epochs is 62 hours. The final model accuracy and loss plots are presented in Fig. 7. The plots depict the performance climbs till around 125 epochs and then plateaus. Although a little overfitting is observed in the last 50 epochs, the trained model is acceptable for the classification task at hand.

### Detection of the degradation mechanism

The evaluation results on test data demonstrate good performance as shown in Table 3. The final pixel-level prediction accuracy achieved by the trained model is 92.8% with mean IU of 72.5%. Table 3 also lists the per-class accuracy for class 0: background. The background class has been introduced to handle inaccuracies of the manual annotation. The accuracy for this class is relatively low, however, it does not affect the overall performance of the model.

**Table 3:** Class accuracy of final architecture of the network.

Class	Per-Class Accuracy
0. Background	47.1%
1. No-cracking	96.4%
2. Parallel	94.8%
3. Delamination	89.7%
4. Transversal-branching	72.6%
5. Longitudinal-branching	89.7%
6. Mudflat	91.2%
**Pixel Accuracy: 92**.**8%**	**Mean IU: 72**.**5%**

**Figure 8:**
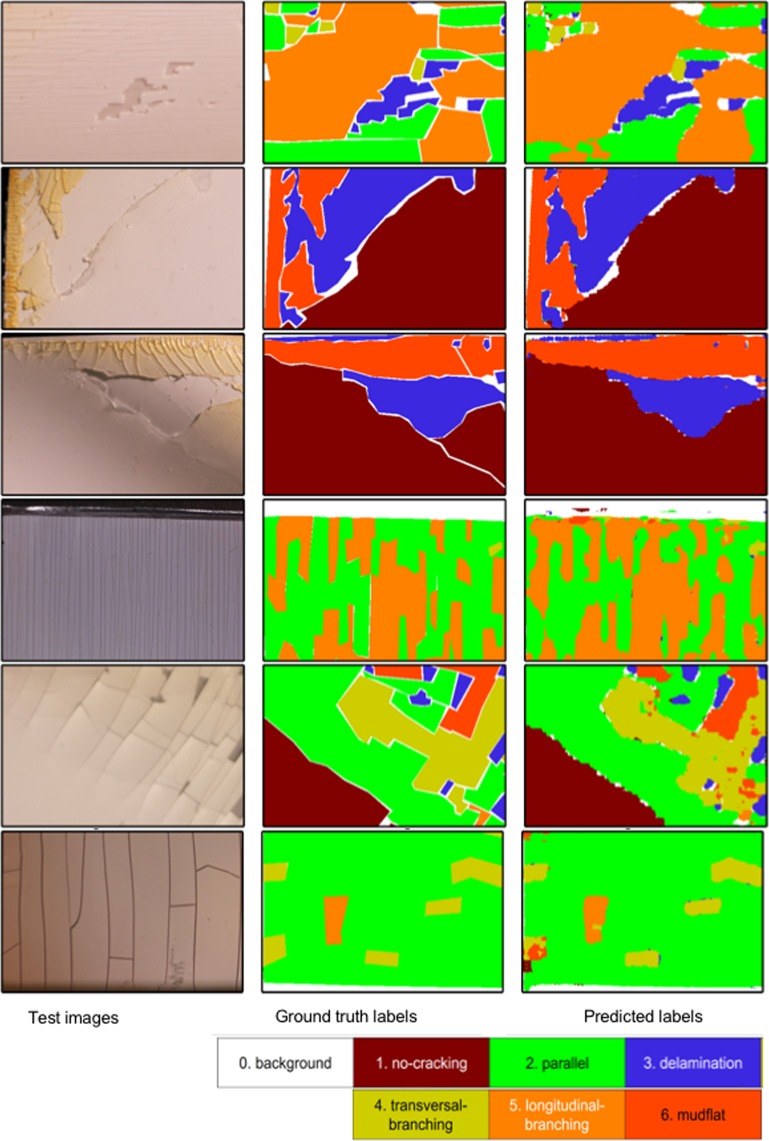
Six examples of crack inspection task performed on the test images (left column) using the trained Model P. The different colors in the middle and right column images indicate different crack classes listed in the legend below.

Detailed results corresponding to the inspection of degradation mechanism in example PV backsheets are depicted in Fig. 8. Three columns in Fig. 8 depict examples of test images, corresponding manually labeled image, and predicted outputs using the trained Model P, respectively. Test images are selected to demonstrate various types of mechanisms that are marked in the legend at the bottom of the figure. The different colors in the second and third columns indicate different crack classes as shown in the color bar in Fig. 8. For example, the test image in the first raw simultaneously exhibits four cracking mechanisms (parallel cracking, delamination, transverse and longitudinal branching cracking). The F-CNN model correctly labeled all mechanisms regardless of different size of individual classes. The predicted crack types and their locations are in good correspondence to the manually labeled classes. All examples depicted in this figure consistently demonstrate the good performance of our model.

In summary, in this work, the utility and efficacy of a fully convolutional neural network architecture for degradation mechanism type detection of PV backsheets has been demonstrated. One of the main contributions of the paper is the development of a F-CNN model that has demonstrated excellent performance in the task of identifying different types of degradation mechanisms in PV backsheets. The pixel level prediction accuracy of the developed F-CNN model is close to 92.8% and the test time per image is 2.1 second. The presented results demonstrate the applicability of the fully-convolutional network in defect detection domain. The proposed architecture is developed by varying the encoding and decoding configuration.

In our framework, we focus on developing a system that can provide high prediction accuracy. Therefore, the evaluation metrics are placed more emphasis than the execution speed in our system. The execution time is also provided as a reference for further improvement of the system.

The developed F-CNN approach is generic and can be adapted to the broad class of segmentation tasks in materials science. Our approach could replace such a manual annotation performed by the microscopy expert to annotate micrographs, or at least suggest the initial annotations. In this sense, our model has an application to any material system, as long as sufficient data is available for model training. The micrograph could also be automatically annotated with the underlying mechanisms, or series of micrographs could be used to construct the entire phase diagram. The initial successes of machine learning in these areas have been recently reported. For example, the micrograph of ultra-high carbon steel has been classified using machine learning^51^. In the same material system - dual phase steel - the damage mechanism has been detected using deep learning^52^. Finally, machine learning has been recently leveraged to construct the phase diagram of low carbon steel^53^.

## Method

F-CNN was developed using Theano (version 1.0.2) and Keras (version 2.2.0). Keras is high-level neural networks application programming interface to enable fast experimentation. Specifically, Keras supports prototyping various convolution neural network architectures. Theano is one of the backend engines for mathematical expression evaluation involving multi-dimensional arrays. The code was developed in Python 2.7.13 and is available on github (https://github.com/Binbin16/Degradation-Mechanism-Detection-By-FCNN). All the experiments in the presented work were conducted on a Linux OS with 12 × 2.66 GHz Intel Xeon X5650 processor cores and 2× Nvidia M2050 Tesla GPUs.
